# Case Report: Recurrent malignant phyllodes tumor with malignant peripheral nerve sheath tumor-like differentiation and loss of H3K27me3/RB1 expression

**DOI:** 10.3389/fonc.2026.1802541

**Published:** 2026-07-28

**Authors:** Meiqi Zhou, Chaonan Li, Jili Qiu, Bingjie Wang

**Affiliations:** 1 Department of Breast Surgery and Oncology, the Second Affiliated Hospital, Zhejiang University School of Medicine, Hangzhou, China; 2 The Key Laboratory of Cancer Prevention and Intervention, China National Ministry of Education, the Second Affiliated Hospital, Zhejiang University School of Medicine, Hangzhou, China; 3 Department of General Surgery, Hangzhou Linping District Integrated Traditional Chinese and Western Medicine Hospital, Hangzhou, China; 4 Department of Pathology, the Second Affiliated Hospital, Zhejiang University School of Medicine, Hangzhou, China

**Keywords:** case report, H3K27me3, heterologous differentiation, malignant peripheral nerve sheath tumor (MPNST), malignant phyllodes tumor

## Abstract

Malignant phyllodes tumor (MPT) is a rare breast neoplasm with high recurrence rates and potential for heterologous sarcomatous differentiation. Malignant peripheral nerve sheath tumor (MPNST)-like differentiation in MPT is exceptionally rare and presents considerable diagnostic and therapeutic challenges. We report a case of a 24-year-old female with recurrent MPT exhibiting MPNST-like differentiation and integrated immunophenotypic and genomic characterization. The patient initially presented in November 2023 with a 3.8 cm right breast MPT treated by wide local excision. Five months later, ultrasound revealed local recurrence (5.8 cm), prompting right mastectomy in April 2024. Sixteen months after mastectomy, a 1 cm right chest wall nodule was identified and completely excised. Pathological examination revealed a high-grade spindle cell malignant neoplasm with MPNST-like morphology, brisk mitotic activity (>10 mitoses/10 HPF), and a Ki-67 index of 80–90%. Immunohistochemistry demonstrated focal S100 positivity, partial SOX10 expression, complete loss of H3K27me3, and absent RB1 protein expression, with cytoplasmic β-catenin positivity supporting phyllodes stromal lineage. A structured differential diagnosis excluded metaplastic spindle cell carcinoma, monophasic synovial sarcoma, fibromatosis/desmoid-type fibromatosis, myofibroblastic sarcoma, undifferentiated pleomorphic sarcoma, and primary *de novo* MPNST. Paired tumor–normal whole-exome sequencing of the recurrent lesion identified a *TP53* truncating mutation (p.R342*) and an *RB1* frameshift mutation (p.S127Vfs*9), the latter concordant with RB1 protein loss by immunohistochemistry. The tumor was microsatellite stable and tumor mutational burden-low, with no reportable copy-number alterations, gene rearrangements or fusions, clinically significant germline variants, or reportable *SUZ12/EED* alterations. These findings support molecular characterization of the recurrent lesion but do not establish clonal continuity with prior tumors or a definitive PRC2-driven mechanism. This case highlights the diagnostic value of integrating serial morphology, immunohistochemistry, and genomic profiling in recurrent MPT with rare MPNST-like differentiation. The combined findings of H3K27me3 and RB1 immunophenotypic loss, *TP53* truncation, and *RB1* frameshift mutation raise the possibility that chromatin-associated and cell-cycle alterations may contribute to neural crest-like heterologous differentiation, although this hypothesis requires further validation.

## 1 Introduction

Phyllodes tumors are uncommon breast neoplasms, with an estimated incidence of 0.5% to 1% (1–3). They are categorized into benign, borderline, and malignant types based on stromal cellularity, atypia, tumor borders, heterologous elements, and stromal overgrowth (4). Malignant phyllodes tumors (MPT) are extremely rare, constituting approximately 10%–25% of all phyllodes tumors, with a global annual incidence of fewer than one case per million individuals (2, 5). Clinically, MPTs often present as painless, rapidly enlarging, firm, and lobulated breast masses, occasionally accompanied by skin tension, thinning, or venous dilation in large tumors (6). They are prone to local recurrence, occurring in up to 30% of cases, and may develop distant metastasis (2, 5, 7–12).

The diagnosis of MPT is challenging because of its histological overlap with primary breast sarcomas, particularly spindle cell sarcomas. In addition, the lack of standardized surgical margin recommendations and the uncertain role of adjuvant therapy complicate clinical management (8, 11, 13–19). Although most MPTs show stromal overgrowth and nuclear pleomorphism, some undergo heterologous sarcomatous differentiation, which may indicate more aggressive tumor biology and influence treatment considerations (20–22). Malignant peripheral nerve sheath tumor (MPNST)-like differentiation is exceedingly rare among these cases and creates considerable diagnostic challenges (19, 23, 24). The molecular basis of this phenotypic evolution remains poorly understood. Loss of H3K27 trimethylation (H3K27me3), a histone modification maintained by Polycomb Repressive Complex 2 (PRC2), is a recognized diagnostic marker in conventional MPNST.

Here we describe a 24-year-old patient with a rapidly recurrent MPT showing MPNST-like differentiation and complete immunohistochemical loss of H3K27me3 and RB1, arising 16 months after mastectomy for high-grade MPT. We integrate serial morphology, immunohistochemistry (IHC), and paired tumor–normal whole-exome sequencing (WES) of the recurrent lesion to discuss the diagnostic approach, differential diagnosis, potential molecular underpinnings, and therapeutic implications.

## 2 Case presentation

### 2.1 Patient information

**Figure 1:**
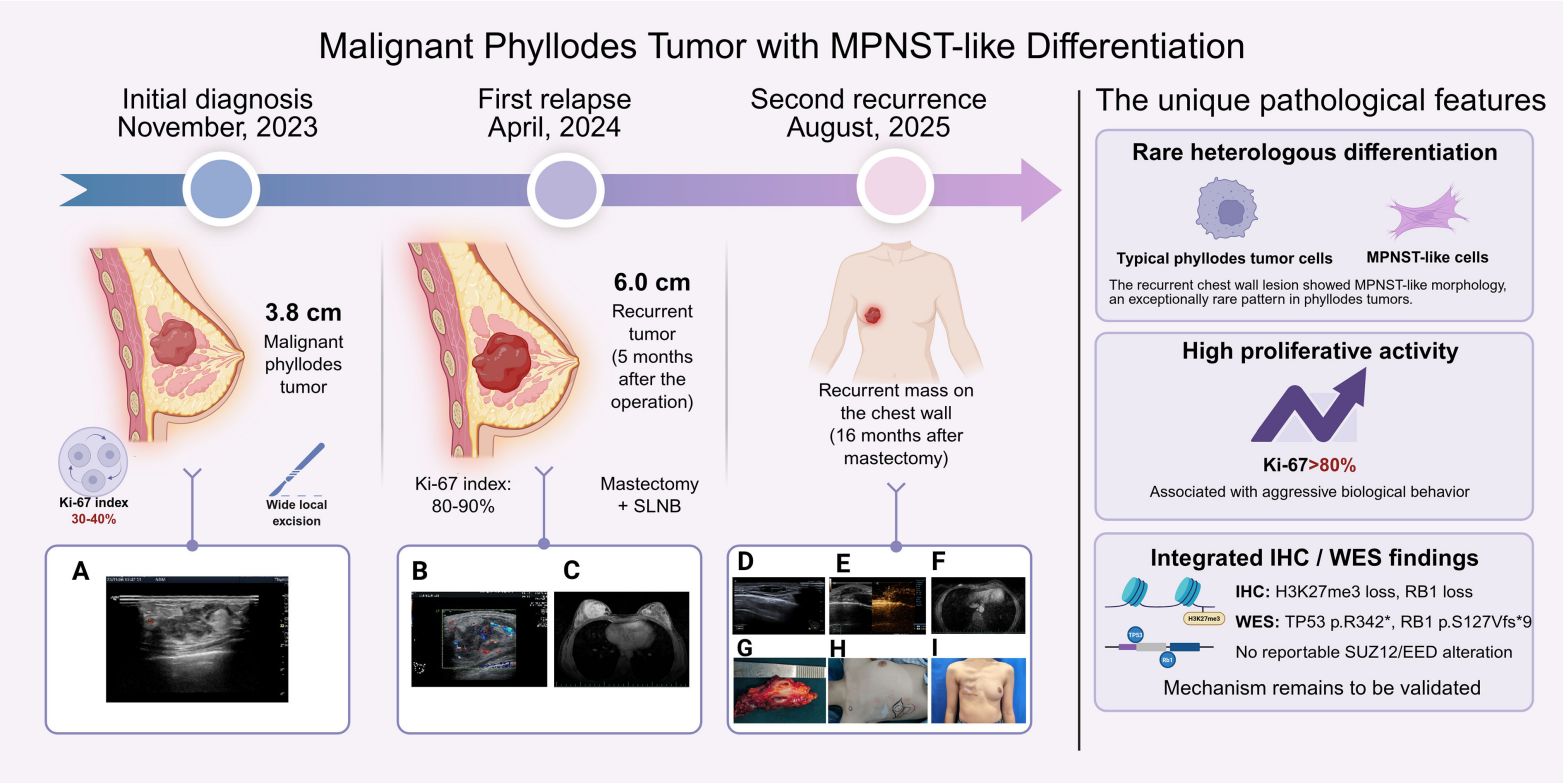
Clinical timeline, imaging findings, surgical course, and integrated immunophenotypic/genomic summary of recurrent malignant phyllodes tumor (MPT) with malignant peripheral nerve sheath tumor (MPNST)-like differentiation. **(A)** Initial breast ultrasound in November 2023 showing an irregular hypoechoic right breast mass with internal vascular signals, subsequently diagnosed as MPT after excision. **(B)** Follow-up ultrasound in April 2024 showing a recurrent complex hypoechoic lesion with abundant vascularity. **(C)** Breast MRI in April 2024 demonstrating an irregular, heterogeneously enhancing right breast mass. **(D, E)** Contrast-enhanced ultrasound in August 2025 detecting an enhancing nodule on the right chest wall. **(F)** Breast MRI in August 2025 confirming a recurrent right chest wall nodule. **(G)** Gross appearance of the resected chest wall specimen. **(H)** Preoperative surgical markings for chest wall tumor resection. **(I)** Well-healed incision at postoperative follow-up. The right schematic summarizes the rare MPNST-like heterologous differentiation, high proliferative activity, and integrated IHC/WES findings in the recurrent chest wall lesion, including H3K27me3 and RB1 loss by IHC, *TP53* p.R342* and *RB1* p.S127Vfs*9 by WES, and no reportable SUZ12/EED alteration. IHC, immunohistochemistry; MPNST, malignant peripheral nerve sheath tumor; MPT, malignant phyllodes tumor; MRI, magnetic resonance imaging; RB1, retinoblastoma protein 1; EED, embryonic ectoderm development; SUZ12, suppressor of zeste 12; SLNB, sentinel lymph node biopsy; WES, whole-exome sequencing.

A 24-year-old female with no significant personal or family history was admitted to the Second Affiliated Hospital, Zhejiang University School of Medicine on August 22, 2025, after noticing a painless right chest wall mass for 5 days. She had a history of right breast MPT. The clinical timeline, imaging findings, surgical course, and key pathological findings are summarized in **Figure 1**.

The patient initially presented to a local hospital in November 2023 with a right breast mass clinically suspected to represent mastitis. Core needle biopsy suggested a mesenchymal tumor of borderline malignancy, and wide local excision was performed. Postoperative pathology confirmed MPT measuring 3.8 × 3.0 × 2.8 cm, with negative margins and no neurovascular invasion (**Figure 1A**). IHC showed ER(−), PR(−), SMA(−), p53 20–30%+, and Ki-67 30–40% (focal hotspot 70–80%).

**Figure 2:**
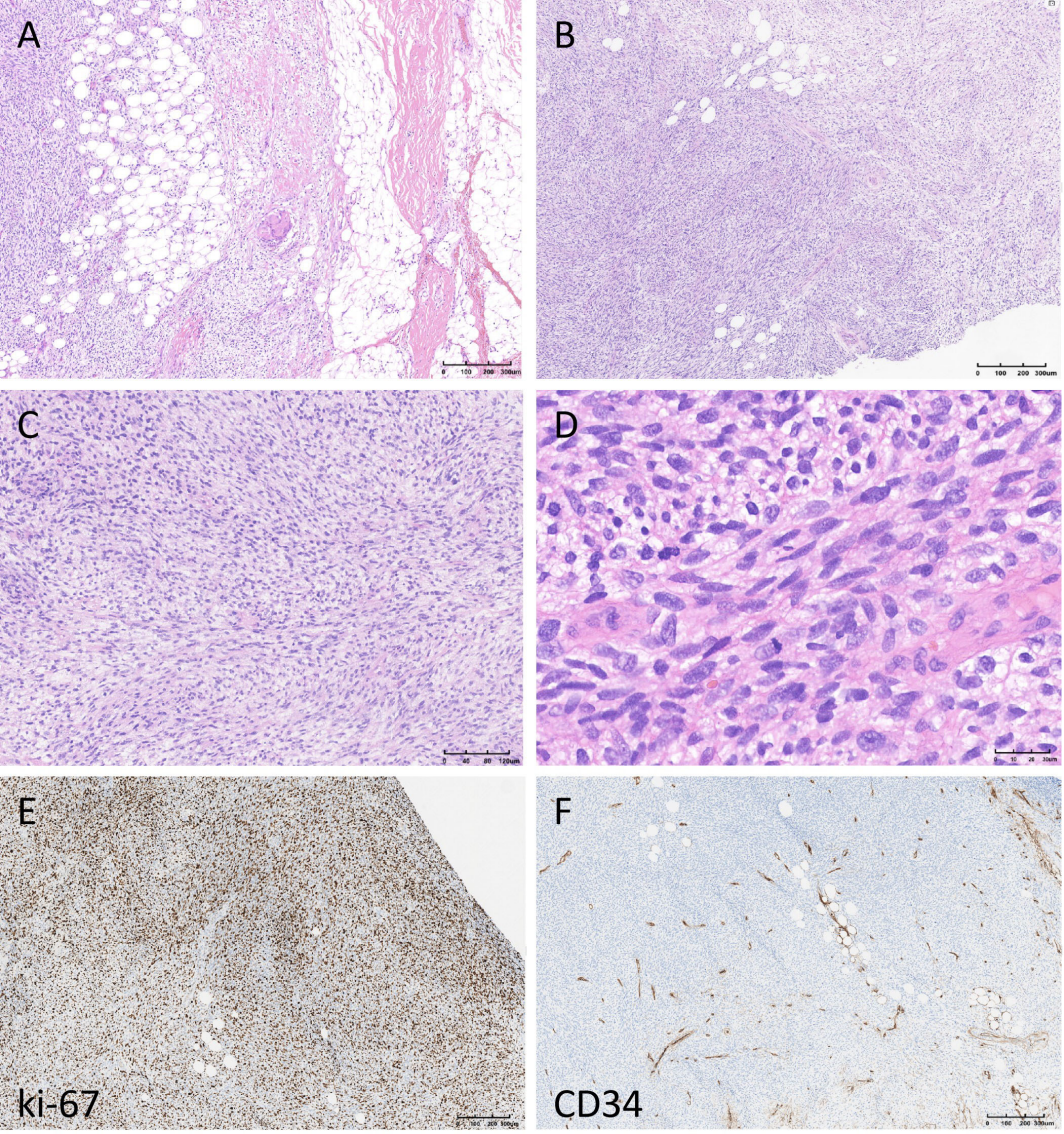
Histopathological and immunohistochemical features of malignant phyllodes tumor (MPT). **(A, B)** MPT showing prominent stromal overgrowth and infiltrative tumor borders, with atypical stromal cells permeating among adipocytes at the tumor periphery (H&E, 40×). **(C)** Stromal atypia with moderate to marked nuclear pleomorphism (H&E, 100×). **(D)** Pleomorphic stromal cells with brisk mitotic activity (H&E, 400×). **(E)** Ki-67 immunohistochemistry showing a high proliferative index in the stromal component (40×). **(F)** CD34 immunohistochemistry showing focal weak stromal expression (40×).

Five months later, follow-up ultrasound on April 19, 2024 revealed a complex cystic-solid right breast lesion (Breast Imaging Reporting and Data System [BI-RADS] 4A; 5.83 × 4.64 × 2.82 cm; **Figure 1B**), and breast magnetic resonance imaging (MRI) showed an irregular heterogeneous enhancement mass (**Figure 1C**). Intraoperative frozen section was consistent with recurrent MPT, and right mastectomy with sentinel lymph node biopsy was performed. All six sentinel lymph nodes were negative. Final pathology showed recurrent MPT measuring 6.0 × 5.0 × 5.0 cm, without vascular or neural invasion (**Figure 2**). IHC showed ER(−), PR(−), HER-2(−), Ki-67 hotspot 80–90%+, p53 40–50%+, CK5/6(−), p63(−), SMA(+). The serial immunophenotypic and molecular evolution is summarized in **Supplementary Table 1**. The multidisciplinary team (MDT) recommended adjuvant chest wall radiotherapy (50 Gy in 25 fractions) and chemotherapy with doxorubicin 60 mg/m² plus cyclophosphamide 600 mg/m² for six cycles. However, the patient declined both treatments.

Sixteen months after the mastectomy, in August 2025, she developed a 1 cm nodule at the right chest wall mastectomy site and was referred to our institution.

### 2.2 Clinical findings

Physical examination showed a well-healed right mastectomy scar with intact overlying skin. A firm, poorly mobile, relatively well-defined 1 cm mass was palpable on the right chest wall. No enlarged axillary or supraclavicular lymph nodes were detected, and the left breast and axilla were unremarkable. Contrast-enhanced ultrasound (**Figures 1D, E**) and breast MRI (**Figure 1F**) demonstrated an enhancing right chest wall nodule measuring 12 × 6 × 14 mm, suggestive of recurrence or metastasis. Core needle biopsy of the chest wall mass confirmed a spindle cell neoplasm favoring recurrent or metastatic MPT. Systemic staging with chest computed tomography showed only scattered pulmonary nodules without definitive evidence of distant metastasis. Abdominal ultrasound and tumor markers were within normal limits.

### 2.3 Diagnostic assessment and therapeutic intervention

**Figure 3:**
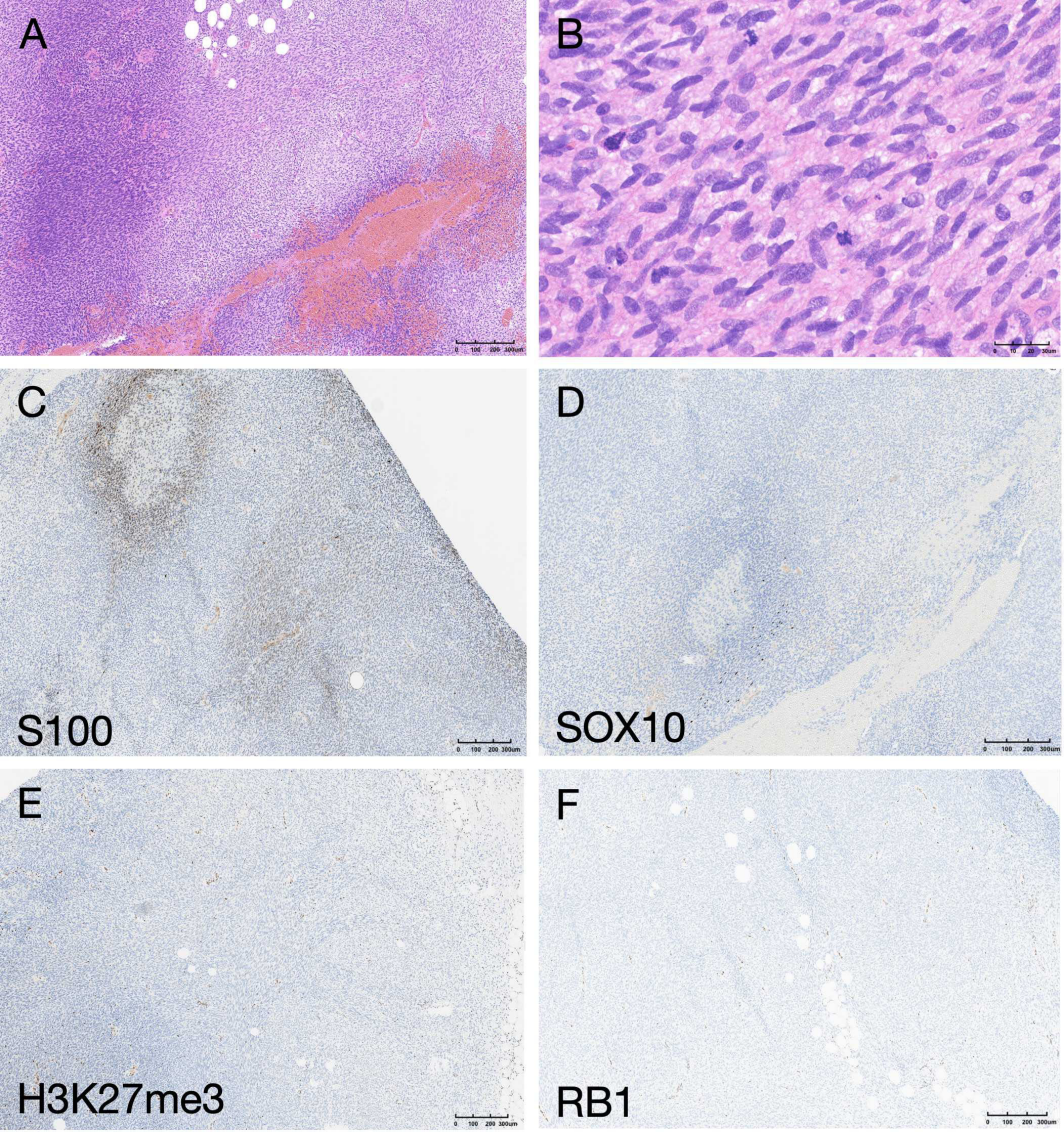
Histopathological and immunohistochemical features of the recurrent chest wall lesion with malignant peripheral nerve sheath tumor (MPNST)-like differentiation. **(A)** Hypercellular spindle cell proliferation involving adipose tissue, with alternating cellular and less cellular areas reminiscent of a marble-like pattern (H&E, 40×). **(B)** High-power view showing atypical spindle cells with wavy nuclei and high-grade MPNST-like morphology (H&E, 400×). **(C)** S100 immunohistochemistry demonstrating focal weak expression in the spindle cell tumor component (40×). **(D)** SOX10 immunohistochemistry showing partial nuclear positivity in tumor cells (40×). **(E)** H3K27me3 immunohistochemistry showing complete loss of nuclear expression in tumor cells, supporting MPNST-like differentiation in the appropriate morphologic and clinical context (40×). **(F)** RB1 immunohistochemistry showing complete loss of nuclear expression in tumor cells (4×).

The patient underwent a wide local excision of the right chest wall tumor with partial pectoralis major muscle resection on August 29, 2025 (**Figures 1G, H**). Histological examination revealed a cellular spindle cell malignant neoplasm with MPNST-like morphology. The tumor showed fascicular and herringbone growth patterns and was composed of atypical spindle cells with hyperchromatic nuclei, conspicuous nucleoli, and moderate to abundant pale cytoplasm. Mitotic figures exceeded 10/10 high-power fields (HPF) (**Figures 3A, B**). No epithelial component was identified and all surgical margins were negative (R0).

IHC was performed on 4-μm formalin-fixed, paraffin-embedded (FFPE) sections using an automated immunostainer (BenchMark ULTRA, Ventana/Roche; antibody details in **Supplementary Table 2**). The tumor showed complete loss of H3K27me3 and RB1 nuclear expression (**Figures 3E, F**), focal S100 positivity, partial SOX10 expression, cytoplasmic β-catenin positivity, Ki-67 80%+, partial CDK4 and c-MYC expression, diffuse p16 expression, and retained INI1/SMARCB1. Tumor cells were negative for CK-PAN, EMA, SMA, desmin, MDM2, STAT6, SS18-SSX, HMB45, ER, PR, CK5/6, p63, and ALK (**Figures 3C–F**).

For integrated molecular characterization, paired tumor–normal WES of the recurrent chest wall lesion was performed using FFPE tumor tissue with matched peripheral blood as germline control. The assay met all quality-control thresholds, with a mean sequencing depth of 1487×, median depth of 1156×, uniformity of 98.56%, mapping rate of 99.94%, Q30 of 95.21%, and paired-sample concordance of 99.97%.

WES identified a *TP53* truncating mutation, c.1024C>T (p.R342*; variant allele fraction [VAF] 68.95%; pathogenic in ClinVar), and an *RB1* frameshift mutation, c.378delC (p.S127Vfs*9; VAF 62.68%; reported as a variant of uncertain significance), the latter biologically concordant with the complete RB1 protein loss seen by IHC. Additional DNA-damage-response variants included *FANCD2* p.K871N and *BMPR1A* p.S365R. No reportable copy-number alterations, gene rearrangements/fusions, or *SUZ12*/*EED* alterations were detected, and no clinically significant germline variants were found. The tumor was microsatellite stable (MSS) with low tumor mutational burden (TMB <1 mutation/Mb; 9.07 percentile). These findings are interpreted in the Discussion.

Following R0 resection, the MDT recommended adjuvant chemotherapy with doxorubicin 60 mg/m² plus cyclophosphamide 600 mg/m² for up to six cycles. The molecular findings were considered exploratory and were not used as standalone treatment determinants.

### 2.4 Follow-up and outcomes

The patient recovered well after surgery, with no recorded perioperative adverse or unanticipated events (**Figure 1I**). At routine follow-up on June 3, 2026, clinical evaluation and available follow-up examinations showed no evidence of local recurrence or distant metastasis. The patient remained under multidisciplinary surveillance. Longer-term oncologic outcomes and treatment adherence will continue to be monitored.

## 3 Discussion

This case of recurrent MPT with rare MPNST-like differentiation highlights key diagnostic and therapeutic challenges. Integrated WES strengthened the molecular characterization of the recurrent lesion but also emphasized the need for cautious, hypothesis-generating interpretation of the immunophenotypic and genomic findings.

### 3.1 Highlights

To our knowledge, complete immunohistochemical loss of H3K27me3 has not been previously documented in MPT with heterologous differentiation. Unlike the more commonly reported osteosarcomatous or liposarcomatous elements in MPT (20–22), the MPNST-like differentiation observed here represents one of the rarest heterologous patterns. Its occurrence in a 24-year-old patient as a chest wall recurrence, rather than a lesion confined to residual breast parenchyma, further underscores the unusual clinicopathological features of this case (2, 15, 25).

### 3.2 Comparison with reported breast MPNST

To contextualize our findings, we reviewed 21 reported cases of breast MPNST with adequate clinicopathological detail (**Table 1**) (26–46), and the details of the literature review are provided in **Supplementary Table 3**. Breast MPNST is exceedingly rare, accounting for under 0.1% of malignant breast tumors. Most reported cases represent primary, *de novo* breast MPNSTs, whereas the present case shows secondary MPNST-like heterologous differentiation arising in a recurrent MPT. Two additional cases represented metastases to the breast from extramammary MPNST (38, 39). This distinction between *de novo* neural sheath malignancy and heterologous differentiation within an established phyllodes lineage has important diagnostic and prognostic implications and underscores the value of serial pathological reassessment in recurrent MPT.

**Table 1:** Clinicopathological summary of 21 reported cases of breast malignant peripheral nerve sheath tumor (MPNST).

Parameter	Findings
Age (years)	Range: 4–80 years Median: 36 years; mean: 40.2 years
Gender	Female: 19 (90.5%) Male: 2 (9.5%)
NF1 Association	NF1-associated: 5 (23.8%) Sporadic/non-NF1: 16 (76.2%)
Location	Left breast: 10 (47.6%) Right breast: 11 (52.4%)
Tumor Size (cm)	Available in 19 cases; range: 2.0–29.0 cm Mean: 9.6 cm; median: 6.0 cm; tumors >10 cm: 7/19 (36.8%)
Common Presentation	Most cases presented as a rapidly growing breast mass Other presentations included pain, ulceration, recurrent lumps, bloody nipple discharge, axillary lymphadenopathy, and inflammatory changes mimicking mastitis
Diagnostic IHC Profile	Frequently positive: S-100, vimentin Other neural or supportive markers: NSE, neurofilament, Nestin, Leu-7 (positive in selected cases) Additional markers, including SOX10, GFAP, HMB45, and H3K27me3, were assessed only in selected cases and showed variable results; H3K27me3 showed complete loss in one conventional high-grade breast MPNST and retained expression in one epithelioid MPNST
Primary Surgical Management	Mastectomy: 10 (47.6%) Wide/local excision or re-excision: 9 (42.9%) No definitive surgery/refused treatment/not specified: 2 (9.5%)
Adjuvant Therapy	Radiotherapy administered or recommended/referred: 11 (52.4%) Chemotherapy: 3 (14.3%)
Clinical Outcome	No residual disease/no recurrence reported: 11 (52.4%) Recurrence or metastasis without reported disease-specific death: 2 (9.5%) Disease-specific mortality: 2 (9.5%) Outcome not reported or unclear: 6 (28.6%)

GFAP, glial fibrillary acidic protein; H3K27me3, trimethylation of histone H3 lysine 27; IHC, immunohistochemistry; MPNST, malignant peripheral nerve sheath tumor; NF1, neurofibromatosis type 1; NSE, neuron-specific enolase; SOX10, SRY-box transcription factor 10.

The reviewed cases showed a marked female predominance (19/21, 90.5%), a substantial neurofibromatosis type 1 (NF1)-associated subset (5/21, 23.8%), and generally large tumor size among cases with available measurements (mean 9.6 cm; median 6.0 cm). S-100 and vimentin were the most frequently reported positive markers, whereas other neural or supportive markers were reported only in selected cases. Treatment was primarily surgical, often requiring wide excision or mastectomy, whereas the roles of adjuvant radiotherapy and chemotherapy remain undefined and are generally considered for large, margin-positive, recurrent, or high-risk tumors (2, 8, 10, 11, 13, 15, 18, 47). No residual disease or no recurrence was reported in 11 cases (52.4%), although follow-up duration and reporting were heterogeneous. Recurrence, metastasis, and disease-related mortality were still observed, supporting the aggressive potential of this entity. Reported prognostic factors include tumor grade, margin status, NF1 association, and radiation-induced origin (26, 31, 35, 38, 39, 41, 42, 45, 46, 48). Radiation-induced cases have a notably poorer prognosis and significantly reduced survival compared to those associated with NF1 or sporadic occurrence (23).

### 3.3 Diagnostic pitfalls

Initial diagnosis of MPT remains challenging, particularly in young patients, in whom rapidly enlarging breast masses may be clinically or radiologically mistaken for mastitis because of atypical imaging features (46). Core needle biopsy may also be insufficient for definitive classification, with final diagnosis often relying on complete postoperative histopathological assessment. Machine learning-based radiomics and deep-learning approaches may help reduce such early diagnostic errors in the future (17, 49).

The greater diagnostic challenge arose at recurrence, where distinguishing MPNST-like heterologous differentiation within a recurrent MPT from a *de novo* chest wall sarcoma required systematic exclusion of multiple mimics (see Differential Diagnosis). In this case, the most informative findings were the prior history of recurrent MPT, cytoplasmic β-catenin positivity supporting phyllodes stromal lineage, focal S100 and partial SOX10 expression, and complete H3K27me3 loss by IHC. Therefore, recurrent spindle cell lesions in patients with a history of MPT should be evaluated with a broad IHC panel that includes epithelial, myogenic, melanocytic, neural crest, and phyllodes-associated markers, interpreted in the context of serial pathology.

### 3.4 Differential diagnosis

The diagnosis was established through structured exclusion of the principal mimics by integrating clinical history, serial morphology, IHC, and available WES findings.

1. Primary *de novo* MPNST of the breast/chest wall was considered less likely because of the patient’s prior pathologically confirmed MPTs at the same anatomical site in 2023 and 2024, cytoplasmic β-catenin positivity supporting phyllodes stromal lineage, and the absence of neurofibromatosis or a pre-existing neural lesion. However, absolute exclusion would require side-by-side molecular clonality testing of the serial specimens, which was not performed. WES of the recurrent lesion strengthened its molecular characterization but could not, by itself, definitively resolve clonal continuity with the earlier MPTs.
2. Metaplastic spindle cell carcinoma was excluded by complete negativity for CK-PAN and EMA, together with the absence of any epithelial component.
3. Monophasic synovial sarcoma was considered unlikely because SS18-SSX expression was negative by IHC. Consistently, WES detected no gene rearrangement or fusion within the captured regions; however, because exome-based assays are not optimized to detect all structural rearrangements, this finding should be interpreted as supportive rather than definitive.
4. Fibromatosis/desmoid-type fibromatosis was excluded by the markedly elevated mitotic rate (>10/10 HPF), high Ki-67 index of 80–90%, and absence of nuclear β-catenin accumulation.
5. Undifferentiated pleomorphic sarcoma was considered less likely because focal S100 and partial SOX10 expression suggested neural crest-like differentiation, which is not typical of this entity.
6. Myofibroblastic sarcoma was argued against by negativity for SMA, desmin, and ALK.

### 3.5 Relationship between the serial lesions

A central question is whether the 2025 chest wall lesion represented recurrence of the original MPT with MPNST-like differentiation or an independent second sarcoma. Several findings favored recurrence: the lesion arose at the same anatomical site after two prior pathologically confirmed MPTs in 2023 and 2024; all lesions shared a stromal/spindle cell phenotype with persistently high proliferative activity; and cytoplasmic β-catenin positivity supported phyllodes stromal lineage. Nevertheless, because the serial specimens were not sequenced side by side, WES of the recurrent lesion could not independently establish clonal identity with the earlier tumors. We therefore interpret recurrence with MPNST-like heterologous differentiation as the most parsimonious explanation based on clinical, morphologic, and immunophenotypic continuity, while acknowledging that definitive proof of clonal continuity would require paired molecular analysis of all serial lesions.

### 3.6 Integrated molecular profiling

Phyllodes tumorigenesis may follow a *MED12*-dependent fibroadenoma-to-phyllodes pathway or a more aggressive *MED12*-independent pathway involving alterations in *EGFR*, *RB1*, *TP53*, and *TERT* promoter status, which is associated with more aggressive clinical behavior and poorer outcomes. In high-grade tumors, the concurrent presence of *TERT* promoter mutations and *TP53* alterations have been linked to chemotherapy resistance and proposed as potential predictive biomarkers (1, 12). In the recurrent lesion, WES identified a *TP53* truncating mutation (p.R342*; VAF 68.95%) and an *RB1* frameshift mutation (p.S127Vfs*9; VAF 62.68%), consistent with a high-grade, *MED12*-independent molecular profile. The *RB1* frameshift provided a plausible genomic correlate for complete RB1 protein loss by IHC.

No reportable *SUZ12* or *EED* alteration was detected, leaving H3K27me3 loss as an immunophenotypic finding without an established genomic basis in this case. Because complete H3K27me3 loss is reported in 69–95% of sporadic or radiation-induced MPNSTs (50–52), it supports MPNST-like differentiation but does not prove a PRC2-driven mechanism. H3K27me3 and RB1 immunophenotypic loss, together with *TP53* and *RB1* sequence variants, raise the possibility that chromatin-associated and cell-cycle alterations may contribute to this rare neural crest-like differentiation.

The tumor was MSS and TMB-low (<1 mutation/Mb), with no reportable copy-number alterations, gene rearrangements/fusions, clinically significant germline variants, or canonical MPNST-associated alterations involving *NF1*, *CDKN2A*, *SUZ12*, or *EED* (50–52). Future studies incorporating DNA methylation profiling, broader structural-variant detection, and circulating tumor DNA monitoring may improve diagnostic precision and postoperative surveillance.

### 3.7 Therapeutic considerations

Early detection of a small chest wall recurrence enabled R0 resection without extensive reconstruction. Complete surgical removal with clear margins remains the definitive foundation for treating localized MPT. Although a 1 cm margin is generally recommended, several studies suggest that guaranteed negative margins may be sufficient in selected cases (18, 53, 54). Mastectomy is generally reserved for large, multiply recurrent, or margin-compromised tumors, and routine axillary surgery is not recommended because MPT metastasizes predominantly through hematogenous routes (8, 53).

The role of adjuvant therapy remains controversial. MPT is relatively insensitive to conventional chemotherapy and radiotherapy, particularly in metastatic settings (1, 12, 13, 55). However, doxorubicin-plus-cyclophosphamide may be considered for high-risk tumors with stromal overgrowth or heterologous sarcomatous components (5, 56), and adjuvant radiotherapy may reduce local failure in selected high-risk cases, although supporting evidence is mainly retrospective (16). Therefore, the patient’s decision to decline adjuvant therapy after the 2024 mastectomy should be interpreted cautiously as only one possible contributor to later recurrence.

The WES findings inform but do not determine systemic treatment. The *TP53* p.R342* mutation was linked in the laboratory report to possible sensitivity to the WEE1 inhibitor adavosertib, but this remains an exploratory, clinical-trial-relevant observation rather than a standard recommendation. The MSS/TMB-low profile and absence of actionable copy-number or fusion events provide no established biomarker supporting immune-checkpoint inhibition. Recent preclinical advances, including establishment of MPT-derived xenograft models demonstrating pazopanib sensitivity and immune modulation strategies, suggest that targeted approaches may represent a promising future direction (1, 11). Thus, systemic therapy should remain MDT-directed and, when appropriate, clinical-trial-oriented rather than driven by a single molecular finding.

The high Ki-67 index, rising to 80%–90% at recurrence, further supports close surveillance and individualized multidisciplinary management (53). A study in a US cohort showed that histology scores were more accurate than Singapore nomograms in predicting recurrence, suggesting that existing prediction models require global collaboration and optimization for more malignant cases (57).

### 3.8 Limitations and future perspectives

This report is limited by its single-case nature and the lack of parallel molecular analysis of the serial 2023, 2024, and 2025 specimens. Although the clinical course, morphology, and immunophenotype favor recurrent MPT with MPNST-like differentiation, clonal continuity could not be definitively proven. The genomic findings from the recurrent lesion should also be interpreted cautiously, as no DNA methylation profiling or functional validation was performed, and no reportable *SUZ12*/*EED* alteration was detected. The proposed links among H3K27me3 loss, *RB1* inactivation, *TP53* alteration, and neural crest-like differentiation remain hypothesis-generating. The molecular treatment implications are exploratory, and longer follow-up is needed. Future studies integrating serial tumor sequencing, methylation profiling, and functional models are needed to clarify the biology and clinical relevance of this rare differentiation pattern.

## 4 Conclusion

This case illustrates the diagnostic complexity of recurrent MPT with rare MPNST-like heterologous differentiation and highlights the value of integrating serial morphology, broad IHC, and genomic profiling. In this case, IHC results of H3K27me3 and RB1 loss, focal S100/SOX10 expression, and β-catenin positivity may help recognize phenotypic divergence from an established phyllodes lineage. The accompanying *TP53* truncation and *RB1* frameshift mutation raise a testable hypothesis that chromatin-associated and cell-cycle alterations may contribute to neural crest-like differentiation, although further genomic, epigenetic, and functional validation is required. The aggressive recurrence pattern supports individualized multidisciplinary management and close surveillance.

## 5 Patient perspective

The patient described the experience of three surgeries within 22 months as physically and emotionally challenging. She reported distress after the rapid recurrence following the initial excision and stated that concerns about treatment-related toxicity contributed to her decision to decline adjuvant therapy after mastectomy. After the chest wall recurrence and subsequent molecular assessment, she expressed a clearer understanding of the aggressive behavior of the disease and the rationale for close surveillance. She indicated willingness to follow the multidisciplinary treatment plan and attend scheduled follow-up.

## Data Availability

The original contributions presented in the study are included in the article/**Supplementary Material**. Further inquiries can be directed to the corresponding authors.
