# Supplementary material for: Case Report: Recurrent malignant phyllodes tumor with malignant peripheral nerve sheath tumor-like differentiation and loss of H3K27me3/RB1 expression
Source: Front Oncol. 2026 Jul 28;16:1802541. doi: 10.3389/fonc.2026.1802541 (PMC13457062; doi:10.3389/fonc.2026.1802541)
Supplement: Supplementary file 1 [file Table1.docx]

**Supplementary Table 1. Serial clinicopathological, immunohistochemical, and molecular evolution of the case.**

Serial clinicopathological, immunohistochemical, and molecular findings from the initial malignant phyllodes tumor, recurrent breast lesion, and recurrent chest wall lesion. Data are summarized from the final manuscript. Molecular testing was performed on the recurrent chest wall lesion only.

| **Time point** | **Specimen / anatomic site** | **Procedure** | **Tumor size** | **Pathological diagnosis / morphology** | **Margins and invasion** | **Immunohistochemistry** | **Molecular findings** | **Clinical management / follow-up** |
| --- | --- | --- | --- | --- | --- | --- | --- | --- |
| November 2023 | Right breast mass | Wide local excision | 3.8 × 3.0 × 2.8 cm | Malignant phyllodes tumor (MPT). | Negative margins; no neurovascular invasion reported. | ER(−), PR(−), SMA(−), p53 20–30%+, Ki-67 30–40% with focal hotspot 70–80%. | Not performed / not available. | Initial local excision. |
| April 2024 | Recurrent right breast lesion | Right mastectomy with sentinel lymph node biopsy | 6.0 × 5.0 × 5.0 cm | Recurrent malignant phyllodes tumor. | No vascular or neural invasion; all six sentinel lymph nodes negative. | ER(−), PR(−), HER2(−), Ki-67 hotspot 80–90%+, p53 40–50%+, CK5/6(−), p63(−), SMA(+). | Not performed / not available. | MDT recommended chest wall radiotherapy (50 Gy/25 fractions) and doxorubicin plus cyclophosphamide for six cycles; patient declined both treatments. |
| August 2025 | Right chest wall nodule at mastectomy site | Wide local excision with partial pectoralis major muscle resection | 12 × 6 × 14 mm on imaging; clinically approximately 1 cm | Cellular spindle cell malignant neoplasm with MPNST-like morphology, fascicular and herringbone growth patterns, >10 mitoses/10 HPF, and no epithelial component identified. | Negative margins (R0). | Complete loss of H3K27me3 and RB1 nuclear expression; focal S100(+), partial SOX10(+), cytoplasmic β-catenin(+), Ki-67 80%+, partial CDK4(+), diffuse p16(+), partial c-MYC(+), retained INI1/SMARCB1; negative for CK-PAN, EMA, SMA, desmin, MDM2, STAT6, SS18-SSX, HMB45, ER, PR, CK5/6, p63, and ALK. | Paired tumor–normal WES identified TP53 c.1024C>T (p.R342*) and RB1 c.378delC (p.S127Vfs*9). No reportable copy-number alterations, gene rearrangements/fusions, SUZ12/EED alterations, or clinically significant germline variants were detected. MSS; TMB-low (<1 mutation/Mb). | MDT-directed adjuvant systemic therapy and close surveillance were recommended. Follow-up on June 3, 2026 showed no evidence of recurrence or distant metastasis according to the final manuscript. |

*Abbreviations: ALK, anaplastic lymphoma kinase; CK, cytokeratin; EMA, epithelial membrane antigen; ER, estrogen receptor; HPF, high-power fields; IHC, immunohistochemistry; MDT, multidisciplinary team; MDM2, mouse double minute 2 homolog; MPNST, malignant peripheral nerve sheath tumor; MPT, malignant phyllodes tumor; MSS, microsatellite stable; PR, progesterone receptor; R0, microscopically margin-negative resection; SMA, smooth muscle actin; TMB, tumor mutational burden; WES, whole-exome sequencing.*
