# Supplementary material for: Case Report: Recurrent malignant phyllodes tumor with malignant peripheral nerve sheath tumor-like differentiation and loss of H3K27me3/RB1 expression
Source: Front Oncol. 2026 Jul 28;16:1802541. doi: 10.3389/fonc.2026.1802541 (PMC13457062; doi:10.3389/fonc.2026.1802541)
Supplement: Supplementary file 2 [file Table2.docx]

**Supplementary Table 2. Primary antibodies used for immunohistochemistry.**

Primary antibody information for immunohistochemical staining reported in the final manuscript. Antibody details were transcribed and standardized from the uploaded institutional IHC antibody list. Staining was performed on formalin-fixed, paraffin-embedded sections using an automated immunostainer as described in the manuscript.

| **Marker / antibody** | **Vendor / catalog number** | **Host species** | **Clone** | **Use in final manuscript** |
| --- | --- | --- | --- | --- |
| ALK | Beijing Zhongshan Golden Bridge; ZM-0848 | Mouse | 1A4 | Negative in recurrent chest wall lesion |
| ALK | Beijing Zhongshan Golden Bridge; ZM-0248 | Mouse | OTI1H7 | Negative in recurrent chest wall lesion |
| c-MYC (MYC) | Beijing Zhongshan Golden Bridge; ZA-0555 | Rabbit | EP121 | Partial expression in recurrent chest wall lesion |
| CDK4 | Beijing Zhongshan Golden Bridge; ZA-0614 | Rabbit | EP180 | Partial expression in recurrent chest wall lesion |
| Cytokeratin, AE1/AE3 | Beijing Zhongshan Golden Bridge; ZM-0069 | Mouse | AE1/AE3 | Epithelial marker; negative as CK-PAN/CK panel in recurrent lesion |
| Cytokeratin, 5/6 | Beijing Zhongshan Golden Bridge; ZM-0313 | Mouse | OTI1F8 | Negative in 2024 lesion and recurrent chest wall lesion |
| Desmin | Beijing Zhongshan Golden Bridge; ZA-0610 | Rabbit | OTIR4A8 | Negative in recurrent chest wall lesion |
| EMA | Beijing Zhongshan Golden Bridge; ZM-0095 | Mouse | GP1.4 | Negative in recurrent chest wall lesion |
| ER | Ventana/Roche | Rabbit | SP1 | Negative in serial lesions / recurrent chest wall lesion |
| H3 K27me3 | Beijing Zhongshan Golden Bridge; ZA-0327 | Rabbit | RM175 | Complete loss of nuclear expression in recurrent chest wall lesion |
| INI-1 | Beijing Zhongshan Golden Bridge; ZA-0696 | Rabbit | OTIR4G9 | Retained INI1/SMARCB1 expression in recurrent chest wall lesion |
| Ki67 | Beijing Zhongshan Golden Bridge; ZM-0167 | Mouse | MIB-1 | Proliferation index in serial lesions |
| MDM2 | Beijing Zhongshan Golden Bridge; ZM-0425 | Mouse | 1E6&17B3 | Negative in recurrent chest wall lesion |
| Melanoma (HMB45) | Beijing Zhongshan Golden Bridge; ZM-0187 | Mouse | HMB45 | Negative in recurrent chest wall lesion |
| P16 | Beijing Zhongshan Golden Bridge; ZM-0205 | Mouse | 1C1 | Diffuse expression in recurrent chest wall lesion |
| S-100 | Beijing Zhongshan Golden Bridge; ZA-0225 | Rabbit | POLY | Focal positivity in recurrent chest wall lesion |
| SOX10 | Beijing Zhongshan Golden Bridge; ZA-0624 | Rabbit | EP268 | Partial positivity in recurrent chest wall lesion |
| SS18-SSX | Cell Signaling | Rabbit | E9X9V | Negative in recurrent chest wall lesion |
| STAT6 | Beijing Zhongshan Golden Bridge; ZA-0647 | Rabbit | EP325 | Negative in recurrent chest wall lesion |
| Rb | Beijing Zhongshan Golden Bridge; ZM-0223 | Mouse | 13A10 | Complete loss of nuclear expression in recurrent chest wall lesion |
| β-catenin | Beijing Zhongshan Golden Bridge; ZM-0442 | Mouse | UMAB15 | Cytoplasmic positivity supporting phyllodes stromal lineage |
| Actin (SMA) | Beijing Zhongshan Golden Bridge; ZM-0003 | Mouse | UMAB237 | Negative in 2023 and recurrent chest wall lesion; positive in 2024 lesion |
| PR | Ventana/Roche | Rabbit | 1E2 | Negative in serial lesions / recurrent chest wall lesion |
| P63 | Beijing Zhongshan Golden Bridge; ZM-0406 | Mouse | UMAB4 | Negative in 2024 lesion and recurrent chest wall lesion |

*Abbreviations: ALK, anaplastic lymphoma kinase; CK, cytokeratin; EMA, epithelial membrane antigen; ER, estrogen receptor; FFPE, formalin-fixed, paraffin-embedded; IHC, immunohistochemistry; PR, progesterone receptor; RB1/Rb, retinoblastoma protein; SMA, smooth muscle actin; SOX10, SRY-box transcription factor 10.*
