# Supplementary material for: Case Report: Recurrent malignant phyllodes tumor with malignant peripheral nerve sheath tumor-like differentiation and loss of H3K27me3/RB1 expression
Source: Front Oncol. 2026 Jul 28;16:1802541. doi: 10.3389/fonc.2026.1802541 (PMC13457062; doi:10.3389/fonc.2026.1802541)
Supplement: Supplementary file 3 [file Table3.docx]

Malignant Peripheral Nerve Sheath Tumors (MPNST) Case Reports

| **Patient Age/ Gender** | **NF1 Status** | **Tumor Location** | **Tumor Size**  **(cm)** | **Symptoms and Presentation** | **Diagnostic Findings (Pathology/IHC)** | **Treatment Administered** | **Clinical Outcome** | **Source** |
| --- | --- | --- | --- | --- | --- | --- | --- | --- |
| 4/F | NF1 positive (Confirmed history) | Left breast (upper outer quadrant) | 2.5 | 3-month history of a painless firm nodule | High-grade MPNST; Positivity for Vimentin and S-100; pleomorphic cells, >5 mitoses/10 HPF, necrosis. | Excisional biopsy, re-excision of 2 cm breast tissue, referred for radiation therapy (RT). | No residual tumor in re-excision specimen. | (1) |
| 27/F | Yes (NF-1) | Right breast (upper outer quadrant - 11 o’clock) | 2.3 | New palpable mass 10 months after treatment for mediastinal MPNST | Metastatic MPNST from mediastinum; Monomorphic spindle cells in interlacing fascicles/whorls; weakly positive for S-100; negative for SMA, Desmin, CD34. | Excision of breast mass, radiation therapy (RT) of right breast. | Expired due to respiratory arrest 24 months after initial mediastinal diagnosis; multiple disseminated metastases. | (2) |
| 36/F | NF1 positive (Confirmed history) | Left breast | Almost the entire breast, five times the size of the right breast | Left breast pain, rapid enlargement; palpable mass for 13 years | Spindle cell sarcoma consistent with MPNST; monomorphic spindled cells with frequent mitotic figures arranged in sweeping fascicles. | Mastectomy | Not in source | (3) |
| 16/F | No (Sporadic) | Left breast (upper inner quadrant) | 11 | Recurrent multiple lumps (five lumps initially) | Spindle cells with alternating hypercellular and hypocellular areas; Weakly positive for S-100; negative for CD10. | Excision, Modified radical mastectomy (MRM), Wide local excision (WLE) for recurrence, IVA chemotherapy (CT) protocol, Radiation therapy (RT). | Surviving well for more than 3 years without locoregional or metastatic recurrence. | (4) |
| 17/M | No (Sporadic) | Lateral aspect of right breast | 6 | Recurrent lump 2 months post-excision; palpable axillary lymph nodes | Epithelioid MPNST; Positive for S-100, CD56, CD99; negative for pancytokeratin; sheets and nests of polygonal cells, mitotic activity 4-5/10 HPF. | Wide local excision (WLE) with nipple areolar complex and breast disc, latissimus dorsi flap reconstruction, adjuvant radiotherapy (50 Gy). | Developed gluteal metastasis 1 month later; palliative wide local excision (WLE). | (5) |
| 22/F | No (Sporadic) | Left breast (upper outer quadrant) | 16 | Rapidly enlarging painless mass for 5 months; fixed and attached to pectoralis major | Spindle cell tumor with whorl formations, brisk mitotic activity; Positive for Vimentin, S-100, Nestin; Ki-67 (20%–30%). | Wide local excision (WLE), adjuvant radiotherapy (RT). | No local recurrence or distant metastasis at 5-year follow-up. | (6) |
| 32/F | No (Sporadic) | Left breast (lower outer quadrant) | 2.5 | Rapidly growing lump for 3 months; history of lumpectomy 10 months prior for neurofibroma | High-grade neurogenic tumor; Vimentin+, S-100+, Neurofilament+, CK-, p63-, SMA-, CD34-, Desmin-. | Wide local excision (WLE), Radiotherapy (RT), Chemotherapy (CT). | No recurrence at 8-month follow-up. | (7) |
| 36/F | No (Sporadic) | Left breast (external quadrants) | 20 | Rapidly growing mass over 1 year; 20 cm size with ulcerative lesion | High-grade spindle cell, epithelioid and pleomorphic sarcoma; positive for SOX-10 and focal S-100; negative for CK CAM 5.2, EMA, SMA, CD10, CD34. | Radical mastectomy, chest wall reconstruction (costal arch resection), adjuvant radiotherapy (60 Gy) and MAI chemotherapy (MAI scheme: doxorubicin, ifosfamide, mesna). | Currently receiving systemic therapy; no hematological toxicity documented. | (8) |
| 38/F | No (Sporadic) | Right breast (upper outer quadrant) | 3.5 | Rapidly growing, painless, hard mass for 3 months | Highly cellular tumor with spindle shaped cells, wavy nuclei, pleomorphic nuclei with abundant mitoses; Positive for NSE, Vimentin, S-100. | Excisional biopsy (advised further treatment) | Not in source | (9) |
| 45/F | No (Sporadic) | Right breast (lower outer quadrant) | 14 | Fast-evolving painful mass, edema, redness, and increased local temperature (simulating mastitis) | High-grade (Grade 3); complete nuclear loss of H3K27me3; diffusely intensely positive for Nestin and Vimentin; negative for S-100, SOX-10, pancytokeratin, CD34; 42 mitoses/10 HPF. | Simple mastectomy including pectoralis muscles and 2 axillary lymph nodes. | Alive with no evidence of disease at 5-month follow-up. | (10) |
| 60/F | No (Sporadic) | Right breast | 26 | Giant lump for 2 years, hidden until ulceration developed; gravitated to groin | Spindle cells with vascular channels; mitotic count 16/10 HPF; positive for NSE, Vimentin, S-100. | Simple mastectomy with excision of axillary node. | Doing well with no local recurrence or distant metastasis at 1-year follow-up. | (11) |
| 65/F | No (Sporadic) | Left breast (upper outer quadrant) | 2.0 | Palpable mass, family history of breast cancer | Low-grade MPNST; moderate pleomorphism, mitoses up to 5/10 HPF; S-100 positive (~20%); negative for AE1/AE3, Cam5.2, p63, GFAP, SMA, Desmin, CD34, HMB-45, SOX-10. | Wide local excision | Well and free of disease 6 months after surgery. | (12) |
| 65/M | No (Sporadic) | Left breast | 2.6 | Rapidly growing, painless, solid fixed mass for 6 months | Melanocytic MPNST; spindled epithelioid cells; strong expression of Vimentin and S-100; pigmented cells positive for HMB-45; negative for CK-8 and NF. | Simple mastectomy, Radiation therapy (RT). | Not in source | (13) |
| 76/F | No (Sporadic) | Left breast (contralateral breast cancer) | 7.5 | Enlarged bilateral breast masses noticed over the past year; left mass elastic, hard, with reddish skin | Epithelioid MPNST; spindle cells with nuclear atypia; positive for Vimentin, S-100, SOX-10, cytokeratin (AE1/AE3), H3K27me3. | Left total mastectomy | No recurrence observed 3 years after surgery. | (14) |
| 80/F | No (Sporadic) | Right breast | 2.7 and 1.9 cm (two masses) | New mass felt 8 months after diagnosis of left renal MPNST | Metastatic MPNST from kidney; spindle cell neoplasm with increased nuclear pleomorphism; positive for S-100; negative for CK cocktail, p63, HMB-45, Melan-A. | Patient refused treatment | Died 6 months later in hospice care. | (15) |
| 33/F | NF1 (multiple café au lait spots and neurofibromas) | Right breast | Not in source | Painful, huge mass growing during pregnancy (4-month history) | Malignant spindle cell findings (consistent with MPNST) | Not in source | Not in source | (16) |
| 19/F | No (Sporadic) | Upper outer quadrant of left breast | 7 | 12-month history of large painless mass, minimal bloody nipple discharge for 3 months | Spindle shape cells, necrosis, pleomorphism, and few mitotic figures; IHC: Vimentin+, S-100+, NSE+, NF+, and GFAP+ | Simple mastectomy and radiotherapy | Not in source | (17) |
| 41/F | Yes (VRN) | Right breast | 20 | 4-year history of recurrent right breast mass; rapid increase in size; multiple cutaneous nodules and café au lait spots | Malignant mesenchymal tumor with spindle cells, rosette-like patterns, palisading; moderate to frequent mitoses. Diagnosis: MPNST in the background of Recklinghausen's disease | Modified radical mastectomy with axillary dissection. (Counseled for but defaulted adjuvant RT/CT) | Local recurrence at 7 months post-operation | (18) |
| 18/F | No (Sporadic) | Upper outer part of right breast | 4 | 2-month history of painless lump | S-100 and Leu-7 focally positive; keratin, CD34, SMA, desmin, GFAP negative; mitotic count 6-10/10 HPF | Excisional biopsy, followed by re-excision of caudal margin (margin initially 0.4 mm). Referred for radiotherapy | No recurrence at 3-year follow-up | (19) |
| 56/F | No (Sporadic) | Entire right breast | 29 | 10-year history of slowly growing mass, became necrotic and ulcerated | Intersecting fascicles of spindle cells; positive for neurofilament, vimentin, S-100, Leu7 | Modified radical mastectomy with axillary dissection and latissimus dorsi flap reconstruction | No evidence of local or distant metastasis at 6-month follow-up | (20) |
| 59/F | No (Sporadic) | Right breast (10 o'clock position) | 3 | 2-month history of painless, firm mass | Spindle cells; S-100 diffusely positive; smooth muscle actin, CD34, CD68 negative; mitotic count 19/10 HPF | Excisional biopsy, followed by wide excision and postoperative radiotherapy | No signs of recurrence at 1-year follow-up | (21) |

The literature search was conducted in PubMed/MEDLINE and Google Scholar using the following search terms: “breast MPNST,” “malignant peripheral nerve sheath tumor breast,” “phyllodes tumor MPNST,” “phyllodes tumor neural differentiation,” and “breast malignant peripheral nerve sheath tumor case report.” The search covered all publications available through January 2026. Inclusion criteria: histologically confirmed breast MPNST or MPT with MPNST-like/neural differentiation, with detailed case-level pathological and clinical information reported. Exclusion criteria: cases lacking sufficient pathological details to allow interpretation; review articles without individual case data; and conference abstracts without peer-reviewed publication. This search strategy identified 21 previously reported cases.

Reference：

1.	Medina-Franco H, Gamboa-Dominguez A, de la Medina AR. Malignant Peripheral Nerve Sheath Tumor of the Breast. *Breast Journal* (2003) 9:332–332. doi: 10.1046/j.1524-4741.2003.09420.x

2.	Park SH, Park JY, Joo M. Imaging Findings of Breast Metastasis from Malignant Peripheral Nerve Sheath Tumor of Mediastinum: A Case Report. *J Korean Soc Radiol* (2023) 84:770. doi: 10.3348/jksr.2022.0116

3.	Bonnet SE, Kang-Chapman JK, Buckley KA, Cui X, Grignol VP, Hawley JR. Malignant peripheral nerve sheath tumor of the breast in a patient with neurofibromatosis 1. *Breast J* (2018) 24:1066–1068. doi: 10.1111/tbj.13127

4.	Shuayb M, Begum R. Unusual primary breast cancer – malignant peripheral nerve sheath tumor: a case report and review of the literature. *J Med Case Reports* (2017) 11:161. doi: 10.1186/s13256-017-1332-1

5.	Suganya S, Ramakant P, Palak R, Paul MJ. Malignant Peripheral Nerve Sheath Tumour of the Male Breast with Nodal Metastasis. *Indian J Surg Oncol* (2015) 6:440–442. doi: 10.1007/s13193-015-0388-x

6.	Chakraborty A, Jalan S, Sarkar DK. Malignant peripheral nerve sheath tumor mimicking breast cancer: a case report. *EJMCR* (2023) 7:164–166. doi: 10.24911/ejmcr/173-1677945890

7.	Agarwal R, Sinha D, Tomar R, Mandal S, Bains L, Jain S. Primary malignant peripheral nerve sheath tumor of the breast. *Breast J* (2019) 25:742–745. doi: 10.1111/tbj.13315

8.	Forero YJ, Zapata Laguado MI, Briceño X, Brugés RE, Contreras F. Peripheral Neural Sheath Breast Sarcoma: Case Report and Literature Review. *Case Rep Oncol* (2022) 15:403–412. doi: 10.1159/000523811

9.	Dhingra KK, Mandal S, Roy S, Khurana N. Malignant peripheral nerve sheath tumor of the breast: case report. *World J Surg Onc* (2007) 5:142. doi: 10.1186/1477-7819-5-142

10.	Val-Bernal J-F, Hermana S, Hernanz F, Moreno G. Primary de novo malignant peripheral nerve sheath tumor of the breast mimicking mastitis. Report of a case and review of the literature. *Rom J Morphol Embryol* (2023) 64:73–82. doi: 10.47162/RJME.64.1.09

11.	Chalkoo M, Ahangar S, Laharwal AR, Patloo AM, Mohd A, Dar SA. Primary Malignant Peripheral Nerve Sheath Tumor of the Breast—A Case Report. *SS* (2011) 02:137–139. doi: 10.4236/ss.2011.23028

12.	Redzepagic J, Skenderi F, Bajrovic J, Beslagic V, Ibisevic N, Vranic S. Low‐grade malignant peripheral nerve sheath tumor: a report of the first case in the breast and literature review. *APMIS* (2016) 124:428–430. doi: 10.1111/apm.12515

13.	Wang H, Ge J, Chen L, Xie P, Chen F, Chen Y. Melanocytic Malignant Peripheral Nerve Sheath Tumor of the Male Breast. *Breast Care* (2009) 4:260–262. doi: 10.1159/000229548

14.	Fukada R, Murakami A, Wada T, Inari H, Yoshida T. Epithelioid Malignant Peripheral Nerve Sheath Tumor of the Breast with Breast Cancer in the Contralateral Breast: A Case Report. *Case Rep Oncol* (2025) 18:531–538. doi: 10.1159/000545496

15.	Rajpurkar A, Koppisetty S, Alessio R. Breast metastases from a malignant peripheral nerve sheath tumor of the kidney: An unusual presentation. *Urol Ann* (2016) 8:387. doi: 10.4103/0974-7796.184878

16.	Tahir M, Zedan M, Bellamkonda V, Dyess DL, Wei EX. Primary Malignant Peripheral Nerve Sheath Tumor of the Breast: A Rare Case Report and Review of Literature. *Cureus* (2022) 14:e31586. doi: 10.7759/cureus.31586

17.	Thanapaisal C, Koonmee S, Siritunyaporn S. Malignant peripheral nerve sheath tumor of breast in patient without Von Recklinghausen’s neurofibromatosis: a case report. *J Med Assoc Thai* (2006) 89:377–379.

18.	Akhator A, Oside CP, Inikori A, Nwanchokor FN. Malignant peripheral nerve sheath tumour: A rare tumour of the breast. Online J Health Allied Scs. 2010;9(1):9. URL: http://www.ojhas.org/issue33/2010-1-9.htm

19.	Elsaify WM, Elsaify MM, Melek RK. De novo malignant peripheral nerve sheath tumor of the breast: case report number one. *Eur Surg* (2007) 39:192–195. doi: 10.1007/s10353-007-0322-8

20.	Woo OH, Yong HS, Lee JB, Kim A, Koo BH, Kang E-Y. A giant malignant peripheral nerve sheath tumour of the breast: CT and pathological findings. *BJR* (2007) 80:e44–e47. doi: 10.1259/bjr/30984808

21.	Yi JM, Moon EJ, Oh SJ, Lee A, Suh YJ, Baek JM, Choi SH, Jung SS. Malignant Peripheral Nerve Sheath Tumor of the Breast in a Patient without Neurofibromatosis: A Case Report. *J Breast Cancer* (2009) 12:223. doi: 10.4048/jbc.2009.12.3.223
